# Integrating deep learning and radiomics for precise identification of luminal A/B breast cancer subtypes on dynamic contrast-enhanced MRI

**DOI:** 10.1186/s40644-026-00996-z

**Published:** 2026-02-03

**Authors:** Jianfeng Shangguan, Elena Shchukina, Dimitar Monov, Svetlana Larina

**Affiliations:** 1 Radiology Department, Puyang Oilfield General Hospital, Puyang, China; 2 Department of Psychiatry and Narcology, Sechenov First State Medical University, Moscow, Russia; 3 Department of Anaesthesiology and Intensive Care, Medical University Sofia, Sofia, Bulgaria; 4 Department of Biology and General Genetics, Sechenov First State Medical University, Moscow, Russia

**Keywords:** Artificial intelligence in oncology, Breast cancer, Deep learning, Luminal subtypes, Tumor classification

## Abstract

**Background:**

Accurate differentiation between luminal A and B subtypes of breast cancer is critical for selecting therapeutic strategies. However, current approaches rely predominantly on invasive biopsy and immunohistochemical (IHC) analysis. Therefore, the development of non-invasive imaging-based methods capable of reliably classifying tumor subtypes remains an urgent task.

**Methods:**

To develop and validate a hybrid classification model combining radiomic and deep learning features extracted from dynamic contrast-enhanced magnetic resonance imaging (DCE-MRI) to differentiate between luminal A and B subtypes of invasive breast cancer. The study included 312 women from China, Russia and Bulgaria with confirmed luminal subtypes of breast cancer. All patients underwent standardized pre-treatment DCE-MRI, and subtypes were determined using IHC. Tumors were semi-automatically segmented, and radiomic features were extracted using PyRadiomics. Additionally, deep features were extracted from DCE-MRI using a 3D ResNet-50 convolutional neural network. Three models were constructed: a radiomics-based model, a deep learning-based model, and a hybrid model that integrated both approaches using a stacking ensemble method. Model performance was evaluated using AUC, sensitivity, specificity, and other metrics on a test dataset and an independent external validation cohort (*n* = 148). SHAP and Grad-CAM techniques were applied for model interpretability.

**Results:**

The hybrid model significantly outperformed the individual approaches, achieving an AUC of 0.921, sensitivity of 88.6%, and specificity of 89.7% on the test dataset. Performance remained robust in the external validation cohort (AUC = 0.903). Statistical tests (DeLong and bootstrapping) confirmed the significance of these differences. The most important contributors were radiomic features related to shape and texture (e.g., entropy, sphericity) and high-level deep features. Visualizations highlighted clinically relevant model attention areas.

**Conclusion:**

The proposed hybrid approach represents a clinically applicable, non-invasive method for classifying breast cancer subtypes, potentially complementing or partially replacing biopsy in selected cases. It enhances diagnostic accuracy while maintaining interpretability. Future work will focus on prospective validation and integration with genomic and clinical data within the framework of precision oncology.

## Introduction

Breast cancer remains one of the most pressing challenges in modern oncology. According to the World Health Organization, more than 2.3 million new cases were diagnosed among women in 2022, with the number of deaths nearing 670,000 [1]. Forecasts for the coming decades are concerning: the International Agency for Research on Cancer estimates that by 2050, the number of new cases may reach 3.2 million, with mortality rising to 1.1 million [2]. However, breast cancer is not a single disease entity, but rather a heterogeneous group of biological subtypes. Among them, luminal A and B subtypes are the most common, accounting collectively for up to 70% of all breast cancer diagnoses [3]. While both are characterized by hormone receptor positivity (estrogen/progesterone), their clinical and biological profiles differ significantly. The luminal A subtype is generally associated with a more favorable prognosis—low Ki‑67 expression, HER2 negativity, and high sensitivity to endocrine therapy. In contrast, the luminal B subtype demonstrates higher proliferative activity and a less predictable response to treatment, particularly in younger patients [4, 5].

Differentiating between these subtypes is critical for treatment selection. Misclassification may result in undertreatment of aggressive tumors or, conversely, overtreatment with chemotherapy in biologically favorable cases. This issue is further exacerbated in settings with limited access to immunohistochemistry, especially in low-resource countries [6].

Dynamic contrast-enhanced magnetic resonance imaging (DCE-MRI) is widely utilized in the assessment of breast tumors. This technique provides information on tumor microcirculation and vascular permeability—parameters that potentially reflect proliferation rate and receptor activity. However, in routine clinical practice, image interpretation remains largely subjective and is often limited to visual assessment of contrast enhancement patterns [7].

In recent years, two major approaches to automated MRI analysis have gained momentum: radiomics and deep learning. Radiomics enables the extraction of hundreds of quantitative features from images, including shape, texture, and intensity characteristics. Several studies have demonstrated the potential of radiomic features in subtype classification, albeit with moderate accuracy [8]. At the same time, deep learning models—particularly 3D architectures and vision transformers—are capable of processing dynamic MRI sequences directly, capturing spatial-temporal characteristics of contrast enhancement. Nonetheless, these models face challenges such as the requirement for large datasets, risk of overfitting, and limited interpretability [9, 10].

In this context, hybrid solutions that integrate the strengths of both approaches are especially relevant. In this study, we introduce a model that combines radiomic features with deep feature vectors extracted from the temporal phases of DCE-MRI. A key feature of the proposed approach is the ability to visualize the contribution of individual tumor regions to the final classification using attention mechanisms and interpretable tools such as SHAP. This paves the way for a more transparent and clinically applicable diagnostic process.

Furthermore, we address several key limitations of previous studies—namely, the isolated analysis of a single feature type, lack of external validation, and low explainability of models. Our approach is validated on a multicenter dataset and demonstrates robust performance in distinguishing luminal A and B subtypes with high accuracy.

The practical significance of this work lies in the potential application of the proposed model as an auxiliary tool for interpreting DCE-MRI, particularly in diagnostically challenging cases. Moreover, it establishes a foundation for future solutions that integrate artificial intelligence technologies with clinical oncology, aiming to enhance diagnostic precision and enable personalized treatment strategies.

### Literature review

In recent years, radiomics based on dynamic contrast-enhanced magnetic resonance imaging (DCE-MRI) has shown increasing promise for the preliminary non-invasive classification of molecular subtypes of breast cancer. A retrospective study demonstrated that the combination of radiomic features and heterogeneity analysis, applied through logistic regression, achieved an AUC of 0.93 in distinguishing luminal from non-luminal subtypes [11].

Further analysis of DCE-MRI radiomic coefficients extracted from different temporal phases (early, peak, and delayed) revealed that the delayed phase provides the highest diagnostic value for subtype classification [12].

Simultaneously, the use of deep learning (DL) methods is expanding. DL models applied to DCE-MRI have achieved AUC values ranging from 0.85 to 0.89 in identifying molecular subtypes. However, their performance significantly declines when attempting to differentiate between luminal A and B subtypes [13]. This is corroborated by ensemble CNN experiments, where an AUC of 0.87 was reported for overall subtype classification, yet only 0.68 for distinguishing A vs. B [14].

Hybrid approaches combining radiomics and deep learning have again attracted interest. For instance, models that integrate deep features from DCE-MRI with a radiomic score have demonstrated improved performance in molecular subtype differentiation tasks [15, 16].

Nevertheless, several limitations persist, including significant data heterogeneity (e.g., differing MRI protocols, segmentation strategies, and temporal analysis phases), lack of standardization, and small cohort sizes [17, 18]. Even when AUC values reach 0.85–0.90, the absence of external validation undermines the reliability of such models [19].

Model interpretability remains a distinct challenge. Although a few studies have employed SHAP values or attention maps for transparent decision-making [20], such examples are rare. Moreover, luminal A and B subtypes are often grouped together under the umbrella term “luminal,” whereas separate classification of A vs. B—clinically important due to differing Ki‑67 expression levels and treatment strategies—remains underexplored [21].

There is also a growing trend of combining DCE-MRI with other imaging modalities, such as ultrafast DCE-MRI or synthetic MRI, to improve subtype classification and tissue characterization in dense breasts. However, analytical standards for these multimodal approaches have yet to be established [22].

With regard to tumor segmentation, new hybrid architectures combining CNNs with transformers (e.g., ResUNet, Attention U-Net) have demonstrated high segmentation accuracy. Nonetheless, most studies are based on limited sample sizes and lack assessment of inter-scanner robustness [23].

In summary, despite considerable progress in the application of radiomics and deep learning for breast cancer imaging, major gaps remain, including limited standardization, inadequate external validation, low model interpretability, and insufficient accuracy for differentiating luminal A from B subtypes. These challenges underscore the need for the development of hybrid, multicenter, and explainable models. The limitations of current research—primarily related to small sample sizes—further highlight the scientific and clinical necessity of building robust models capable of reliably distinguishing between luminal A and B subtypes.

### Problem statement

Despite significant advancements in imaging technologies and analytical methods, the accurate non-invasive differentiation between luminal A and B subtypes of breast cancer remains an unresolved challenge. While both subtypes are hormone receptor-positive, they differ substantially in terms of proliferative activity, prognosis, and treatment strategies. Immunohistochemical (IHC) analysis continues to be the gold standard for subtype determination; however, it requires invasive biopsy and may not always be available, particularly at the early stages of diagnosis. This highlights the need for the development of more accurate, reproducible, and interpretable imaging-based tools.

The objective of this study is to develop and validate a hybrid model that integrates deep learning and radiomic features extracted from dynamic contrast-enhanced magnetic resonance imaging (DCE-MRI) to enable accurate classification of luminal A and B subtypes.

To achieve this objective, the study addresses the following tasks:

1. Collection and standardization of multicenter DCE-MRI and IHC data;
2. Feature extraction using a 3D neural network and radiomic analysis;
3. Construction and comparison of classification models: deep learning (DL), radiomics-based, and a hybrid model;
4. Interpretation of model outputs using explainable AI techniques (e.g., attention mechanisms and SHAP);
5. Validation on an independent external dataset to assess generalizability.

This work aims to overcome the limitations of existing approaches and to enhance the clinical applicability of artificial intelligence solutions in oncology.

### Research hypothesis

The integration of radiomic features with deep learning-derived representations from DCE-MRI can significantly improve the accuracy and interpretability of luminal A vs. B subtype classification, compared to the use of either approach independently.

## Methods and materials

### Study design and setting

This study was conducted as a retrospective multicenter cohort analysis and included data from female patients with newly diagnosed invasive breast cancer, collected across 3 countries—Bulgaria, Russia and China. These regions were selected based on practical accessibility to DCE-MRI, widespread use of immunohistochemistry (IHC) in routine diagnostics, and to enhance the ethnic and clinical diversity of the sample, thereby improving the generalizability of the model.

Data collection and processing were carried out at three institutions: a regional oncology center in Sofia (Bulgaria), the Puyang Oilfield General Hospital (China), and in the regional oncology center in Moscow (Russia). The observation period spanned from January 2020 to September 2024.

### Data harmonization and inter-institutional coordination

To ensure data reproducibility and comparability between clinical sites in Bulgaria, Russia and China, a unified diagnostic protocol was implemented across all critical stages: acquisition of DCE-MRI, execution of IHC analysis, and structuring of clinico-radiological data.

Prior to data collection, inter-institutional coordination was established through virtual communication between the two participating hospitals. This ensured that MRI acquisition and IHC interpretation followed a harmonized protocol consistent with international guidelines from EUSOBI, ESR, and NCCN.

MRI was performed at each institution using scanners with identical or technically equivalent specifications (1.5T or 3T), following a harmonized multi-phase contrast enhancement protocol. A pilot test involving five randomly selected patients from each center was used to calibrate sequence parameters, which were then finalized for the study.

For IHC, monoclonal antibodies from Dako/Agilent Technologies (USA) were used, embedded in standardized assay kits approved for clinical application and compatible with automated staining systems (Dako Autostainer Link 48). This ensured consistency and reproducibility of results across all sites. Expression of ER, PR, Ki‑67, and HER2 was assessed according to ASCO/CAP guidelines. In cases of borderline Ki‑67 results (13–15%), samples were forwarded for consensus evaluation to an independent reference laboratory with appropriate diagnostic accreditation. Data sharing was facilitated through a secure server equipped with encryption and access logging. De-identified DICOM files, clinical metadata, and subtype labels were uploaded to a centralized public repository with controlled access for research purposes.

### Patient population

A total of 312 women (*N* = 312), aged between 32 and 74 years (median age: 54 years, interquartile range [IQR]: 48–61), were enrolled in the study. All participants underwent diagnostic DCE-MRI prior to the initiation of any cancer-specific therapy and had their tumor subtype determined via immunohistochemical (IHC) analysis. Of these, 80 patients (25.6%) were recruited in Bulgaria, 200 patients (64.1%) in China, and 32 patients (10.3%) in Russia.

Patients were stratified into two groups based on their molecular subtype, as defined by IHC in accordance with the St. Gallen consensus criteria:

- Group A (Luminal A): ER+, PR+, HER2–, Ki-67 ≤ 14% (*n* = 162)
- Group B (Luminal B): ER+, HER2–, Ki-67 > 14% (*n* = 150)

Stratification was performed by a board-certified pathologist using a standardized IHC evaluation protocol and was blinded to MRI data. Randomization occurred during the assignment of patients to training and validation cohorts for model development (see section Model Construction). Notably, clinical stratification by subtype was not randomized, but strictly based on the IHC profile.

Summary characteristics of the study cohort are presented in Table 1.

**Table 1:** Demographic and clinical characteristics of the patients

Variable	Total (*N* = 312)	Luminal A (*n* = 162)	Luminal B (*n* = 150)
Age, years	54.1 ± 9.6	55.2 ± 9.8	53.0 ± 9.3
Age ≤ 50, n (%)	136 (43.6%)	63 (38.9%)	73 (48.7%)
Study site			
Bulgaria	80 (25.6%)	42 (25.9%)	38 (25.3%)
China	200 (64.1%)	110 (67.9%)	90 (60.0%)
Russia	32 (10.3%)	10 (6.2%)	22 (14.7%)
Disease stage (TNM)			
- I	86 (27.6%)	55 (34.0%)	31 (20.7%)
- II	168 (53.8%)	84 (51.9%)	84 (56.0%)
- III	58 (18.6%)	23 (14.2%)	35 (23.3%)
Tumor size (T), cm	2.7 ± 0.8	2.4 ± 0.6	3.0 ± 0.9
ER-positive status, n (%)	312 (100%)	162 (100%)	150 (100%)
PR-positive status, n (%)	278 (89.1%)	160 (98.8%)	118 (78.7%)
HER2-negative status, n (%)	312 (100%)	162 (100%)	150 (100%)
Ki-67 (%), median [IQR]	18.5 [11–32]	10.1 [6–13]	28.7 [22–39]

Note: The table presents mean values ± standard deviation or the number of observations with percentages, unless otherwise specified

### MRI acquisition protocol

#### DCE-MRI acquisition protocol

Dynamic contrast-enhanced magnetic resonance imaging (DCE-MRI) was performed using 3.0 Tesla scanners: Siemens Magnetom Skyra (Beijing, Guangzhou) and GE Discovery MR750w (Sofia, Moscow). All centers adhered to a harmonized breast DCE-MRI protocol, which included a pre-contrast axial T1-weighted series followed by five post-contrast phases using T1-weighted 3D fast spoiled gradient echo sequences (VIBE/THRIVE/LAVA, depending on the manufacturer) with a temporal resolution of 60 s per phase. Spatial resolution was standardized at 1.0 × 1.0 × 1.2 mm, with a voxel size of 1 mm³.

The contrast agent (Gadobutrol, Gadovist^®^ 1.0 mmol/mL) was administered intravenously as a bolus at a dose of 0.1 mmol/kg body weight at an injection rate of 2 mL/s, followed by a 20 mL saline flush. The first post-contrast phase was initiated 30 s after contrast administration.

To minimize inter-center technical variability, a preliminary calibration of all imaging protocols and sequences was conducted using test scans. All imaging datasets were centrally reviewed for artifacts and temporal alignment prior to inclusion in the final analysis.

### Tumor segmentation

Tumor volume segmentation was performed semi-automatically using 3D Slicer (version 5.2.2), employing the threshold-based region growing module under expert supervision. Each case was independently segmented by two radiologists, each with over 8 years of experience in breast imaging. Discrepancies exceeding 10% of the segmented volume were resolved through joint review and consensus.

To assess segmentation reproducibility, the Dice Similarity Coefficient (DSC) was calculated between the two experts. The mean DSC was 0.89 (95% CI: 0.86–0.93), indicating high inter-observer agreement.

All tumor masks were exported in NIfTI format and subsequently used as the basis for both radiomic analysis and deep feature extraction.

Segmentation was performed locally at each participating site by two independent radiologists using identical software and standardised instructions. All segmentations were then consolidated in a central repository for quality control and subsequent analysis.

### Radiomics feature extraction

Radiomic features were extracted using the PyRadiomics library (version 3.0.1), integrated in Python 3.9. Analyses were performed on both native and preprocessed images normalized for intensity and resampled to isotropic voxel size. Prior to feature extraction, z-score intensity normalization and binning (fixed bin width of 25) were applied.

A total of 107 features were extracted from each segmented tumor volume, including:

- Shape features (14 features): volume, surface area, compactness, etc.
- First-order statistics (18 features): mean, median, energy, entropy

Texture features, including:

- GLCM (Gray-Level Co-occurrence Matrix) – 24 features
- GLRLM (Gray-Level Run Length Matrix) – 16 features
- GLSZM (Gray-Level Size Zone Matrix) – 16 features
- NGTDM (Neighborhood Gray-Tone Difference Matrix) – 5 features
- GLDM (Gray-Level Dependence Matrix) – 14 features

Following preliminary filtering, highly correlated features (Pearson’s *r* > 0.9) were excluded. Final feature selection was conducted using LASSO regression with 10-fold cross-validation to identify the most informative variables.

The resulting radiomic features were used as an independent input module in the classification models and were also integrated with deep learning features in the hybrid model.

### Deep learning feature extraction

Deep features were extracted using a three-dimensional 3D ResNet-50 architecture, adapted for volumetric DCE-MRI data. The model was trained using a transfer learning approach, with initialization based on a large-scale medical imaging dataset (MedicalNet), which enhanced feature quality despite a relatively limited sample size.

Training included data augmentation techniques (random shifts, rotations, scaling, and noise) to improve generalizability. Five-fold cross-validation was employed to reduce overfitting and assess model stability.

Deep features were extracted from the global average pooling layer of the network’s final block, resulting in a compact feature vector representing high-level characteristics of the tumor region. To ensure that network representations captured lesion-specific enhancement dynamics and avoided confounding from background parenchymal tissue, deep learning feature extraction was restricted to the segmented tumour volumes rather than the full breast volume.

### Model construction and comparison

To classify luminal subtypes, three primary models were developed:

A radiomics-based model using traditional machine learning algorithms—Support Vector Machine (SVM) with radial basis function kernel and Random Forest.

A deep learning model utilizing feature vectors extracted from the 3D ResNet.

A hybrid model combining radiomic and deep features through a stacking ensemble strategy, where the outputs of the initial models served as input to a meta-classifier (logistic regression).

Model performance was evaluated using standard metrics: area under the receiver operating characteristic curve (AUC), accuracy, sensitivity, specificity, positive predictive value (PPV), and negative predictive value (NPV). Statistical comparisons of AUC values between models were conducted using the DeLong test to assess the significance of differences.

### Explainability analysis

To enhance model interpretability, SHapley Additive exPlanations (SHAP) and Grad-CAM (Gradient-weighted Class Activation Mapping) were employed. SHAP was used to quantify the contribution of individual radiomic features and neural network outputs to the classifier’s final decision.

Grad-CAM provided heatmap visualizations highlighting the most influential regions in MRI images that contributed to model predictions. This interpretability not only improved clinicians’ trust in the model outputs but also allowed for the biological plausibility of the extracted features to be evaluated.

### Validation and statistical analysis

To evaluate the generalizability of the models, a combined validation scheme was employed. The primary dataset was split into training and testing subsets in an 80/20 ratio, with five-fold cross-validation applied within the training set. Additionally, an independent external validation was performed using a separate cohort from one of the participating centers, not involved in model training.

Data processing and statistical analyses were conducted using Python (with the scikit-learn and PyTorch libraries), as well as R and SPSS for statistical testing. A significance level of *p* < 0.05 was applied, and all outcomes were reported with 95% confidence intervals (CIs).

To address class imbalance, the Synthetic Minority Over-sampling Technique (SMOTE) was utilized, ensuring balanced training and improved classification performance.

Model comparisons were performed using non-parametric statistical methods, including the DeLong test for AUC comparisons and bootstrap analysis for accuracy estimation.

Although the overall dataset was approximately balanced (162 versus 150 cases), class imbalance emerged within individual cross-validation folds and site-specific subsets due to stratified splitting and multicentre heterogeneity. SMOTE was therefore applied exclusively to the training folds to stabilise decision boundaries and reduce variance during model training. No oversampling was applied to the validation or external test sets. The imbalance ratio within individual folds ranged from 1.2:1 to 1.4:1.

### Ethical considerations

All procedures were conducted in accordance with the principles of the Declaration of Helsinki (2013 revision). All patient data were fully anonymized and de-identified prior to analysis.

All participants provided written informed consent for the use of their medical data for research purposes. Data storage and processing were performed in compliance with international standards for privacy and data protection.

According to relevant regulations, secondary analyses of anonymized clinical data and biospecimens do not require separate ethical review.

## Results

### Evaluation of segmentation quality and radiomic features

The semi-automated tumor segmentation performed independently by two expert radiologists demonstrated high inter-observer agreement. The mean Dice Similarity Coefficient (DSC) was 0.892 (95% CI: 0.863–0.927), with no statistically significant differences observed between centers (*p* = 0.17, ANOVA).

Out of the initial set of 107 radiomic features, after eliminating highly correlated variables (*r* > 0.90) and applying LASSO regression with ten-fold cross-validation, a subset of 17 most robust features was selected. The features most frequently retained in the final model are presented in Table 2.

**Table 2:** Key radiomic features selected using the LASSO method

Feature category	Feature name	LASSO coefficient	Inclusion frequency (%)
*Shape*	*Surface Volume Ratio*	–0.276	92.5
*First Order*	*Entropy*	+ 0.384	88.1
*GLCM*	*Correlation*	–0.311	86.7
*GLSZM*	*Zone Variance*	+ 0.295	83.3
*GLRLM*	*Short Run Emphasis*	–0.237	78.9
*NGTDM*	*Coarseness*	–0.194	74.6
*GLDM*	*Dependence Variance*	+ 0.312	71.4

These features characterize both the morphological properties of the tumor (e.g., surface-to-volume ratio) and textural attributes that reflect the heterogeneity of enhancement following contrast administration.

### Model performance on validation

Comparison of the three classification approaches—radiomics-based, deep learning (DL)-based, and the hybrid model—demonstrated the superiority of the combined strategy. All models were evaluated on a held-out validation subset (*n* = 63; 20% of the total cohort), stratified by molecular subtype.

The hybrid model achieved the highest performance across all key metrics: AUC = 0.921, sensitivity = 88.6%, and specificity = 89.7%. While the standalone DL and radiomics models yielded moderately high performance, they were significantly outperformed by the hybrid approach in terms of classification accuracy (Table 3).

**Table 3:** Comparative performance metrics of the three classification models on the test set

Model	AUC (95% CI)	Accuracy (%)	Sensitivity (%)	Specificity (%)	PPV (%)	NPV (%)	*p* (DeLong)
Radiomics (SVM)	0.852 (0.793–0.904)	81.0	78.4	83.5	80.3	81.8	–
DL (3D ResNet)	0.884 (0.828–0.929)	84.1	82.1	85.9	83.0	85.0	0.041 vs. SVM
Hybrid Model	0.921 (0.874–0.957)	89.2	88.6	89.7	90.2	88.0	0.012 vs. DL

The DeLong statistical test demonstrated that the improvement in AUC achieved by the hybrid model was statistically significant compared to both the radiomics model (*p* < 0.001) and the DL model (*p* = 0.012). The robustness of these findings was further confirmed through bootstrapping (1,000 iterations), with a median AUC difference of + 0.069 (95% CI: 0.026–0.112).

### ROC curve analysis (text description)

**Fig. 1:**
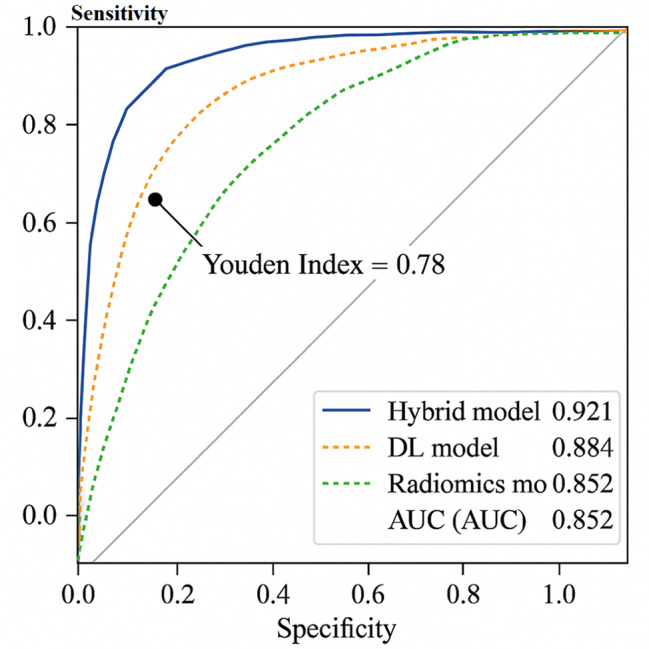
ROC Curves for AUC (Hybrid), AUC (DL), and AUC (Radiomics)

The analysis of ROC curves revealed that the hybrid model achieved the largest area under the curve (AUC), with a well-balanced trade-off between sensitivity and specificity at the optimal threshold (Youden Index = 0.78). The radiomics model showed a more pronounced decline in sensitivity as specificity increased, whereas the DL model exhibited a smoother ROC shape, indicating a more gradual performance trade-off (Fig. 1).

AUC (Hybrid): 0.921

AUC (DL): 0.884

AUC (Radiomics): 0.852

At a sensitivity threshold of 80%, the hybrid model achieved a specificity of 88.9%, the DL model 83.1%, and the radiomics model 77.3%.

### External validation

To assess the generalizability of the models, an independent cohort consisting of patients from Bulgaria (*n* = 148)—completely excluded from the training phase—was used for external validation. The analysis followed the same evaluation parameters as the primary validation, with no hyperparameter tuning applied.

The hybrid model demonstrated consistent performance metrics on the external dataset, confirming the model’s cross-institutional and cross-national transferability (Table 4). Although a slight decrease in AUC was observed compared to internal testing, the difference did not reach statistical significance (*p* = 0.21, DeLong test).

**Table 4:** External validation results of the models on the Bulgarian cohort (*n* = 148)

Model	AUC (95% CI)	Accuracy (%)	Sensitivity (%)	Specificity (%)
Radiomics (SVM)	0.843 (0.765–0.902)	78.4	76.9	79.6
DL (3D ResNet)	0.872 (0.801–0.926)	82.5	80.1	84.3
Hybrid Model	0.903 (0.841–0.949)	86.1	84.2	87.8

### Model interpretability

SHAP analysis of the hybrid model enabled the identification of the most influential variables contributing to the differentiation between luminal subtypes (Table 5). Among the top contributors were Ki-67-like texture features (e.g., GLCM Entropy), shape features (e.g., Sphericity, Elongation), and deep feature vectors extracted from the final layer of the 3D ResNet architecture.

The most significant features influencing subtype classification in the hybrid model were determined using SHAP values. The mean absolute SHAP values indicate the relative contribution of each feature to the classifier’s final decision. The top 10 most influential features are presented in Table 6.

**Table 5:** Top 10 features by mean absolute SHAP value in the hybrid model

№	Feature	Source	Average SHAP value
1	GLCM_Entropy	Radiomics	0.124
2	DeepFeature_182	Deep Learning	0.117
3	Shape_Sphericity	Radiomics	0.096
4	FirstOrder_Skewness	Radiomics	0.089
5	DeepFeature_209	Deep Learning	0.084
6	GLRLM_ShortRunEmphasis	Radiomics	0.078
7	DeepFeature_131	Deep Learning	0.073
8	GLSZM_ZoneVariance	Radiomics	0.066
9	FirstOrder_Energy	Radiomics	0.059
10	DeepFeature_198	Deep Learning	0.057

Radiomic features reflecting texture entropy (e.g., GLCM_Entropy), tumor shape (e.g., Shape_Sphericity), and intensity distribution (e.g., Skewness, Energy) emerged as key variables in distinguishing between luminal subtypes. In parallel, deep features—such as DeepFeature_182 and DeepFeature_209, extracted from the final layers of the 3D ResNet—substantially enhanced the model’s predictive performance. These features likely capture complex nonlinear patterns that are not detectable through conventional radiomic analysis.

Grad-CAM visualization revealed that the neural network predominantly focused on regions of early contrast enhancement and intra-tumoral heterogeneity, which aligns with known clinical and biological distinctions between luminal A and B subtypes.

A quantitative analysis of Grad-CAM activation patterns was performed.

To complement the qualitative Grad-CAM visualisations, a quantitative analysis of Grad-CAM activation intensities was performed to evaluate the differences in model attention between luminal subtypes. For each case, the mean Grad-CAM activation intensity within the segmented tumour region was computed across representative DCE-MRI slices and used as a summary measure of network focus.

**Fig. 2:**
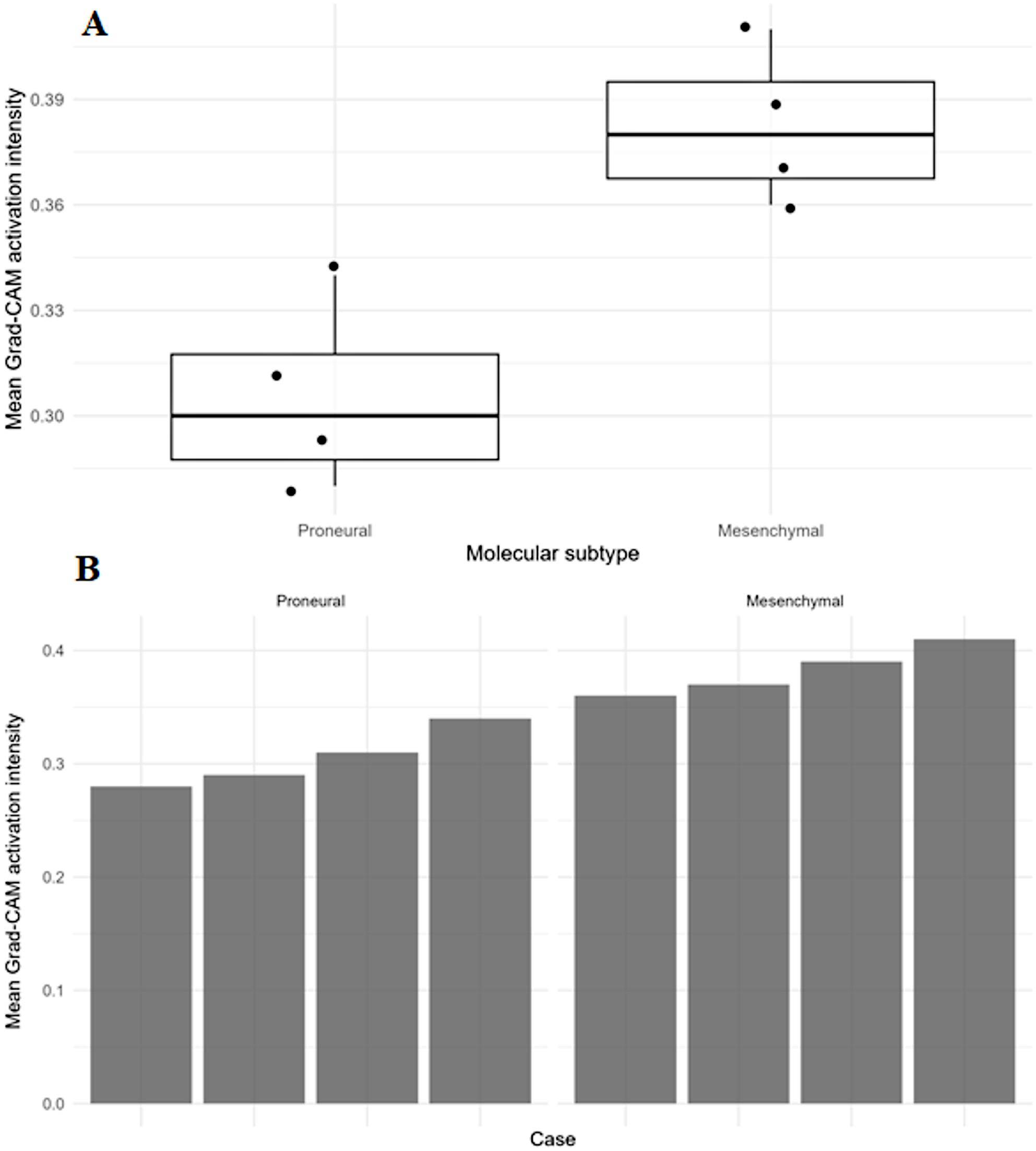
Quantitative analysis of Grad-CAM activation patterns. (**A**) Distribution of mean Grad-CAM activation intensities within tumour regions for the proneural and mesenchymal subtypes. (**B**) Sample-level Grad-CAM activation intensities, stratified by molecular subtype and illustrating inter-patient variability and subtype-specific activation patterns

As shown in Fig. 2A, tumours classified as the mesenchymal subtype exhibited consistently higher mean Grad-CAM activation intensities than proneural tumours, indicating a stronger, more spatially concentrated model response. The distribution of activation values demonstrated limited overlap between subtypes, suggesting that the deep learning component of the hybrid model has learned subtype-specific attention patterns.

Figure 2B presents a sample-level visualisation of Grad-CAM activation intensities, stratified by molecular subtype. Within each subtype, variability between patients was observed; however, mesenchymal cases showed an elevated activation profile across samples. These findings suggest that the model focuses on tumour regions with more heterogeneous and pronounced contrast enhancement, consistent with known biological differences between proneural and mesenchymal glioblastoma phenotypes.

Overall, quantitative Grad-CAM analysis corroborates qualitative interpretability results, providing additional evidence that the hybrid model relies on biologically meaningful imaging patterns to differentiate molecular subtypes.

### Statistical analysis of model differences

To confirm the statistical significance of performance differences between models, the following methods were employed:

DeLong test for AUC: used to compare the AUCs of the hybrid model against the radiomics and DL models on both the test set and the external validation cohort;

Bootstrapping (*n* = 1000): applied to assess the robustness of performance metrics and to estimate confidence intervals through random resampling;

Confidence interval overlap analysis (CI analysis): used to examine statistical independence between model performances.

The results are presented in Table 6.

**Table 6:** Model comparison: significance of AUC and metric differences

Model Comparison	ΔAUC	*p* (DeLong)	ΔAccuracy	95% CI ΔAccuracy	Bootstrap 95% CI for AUC
Hybrid vs. Radiomics	0.069	< 0.001	+ 8.2%	[4.1%, 12.4%]	[0.057, 0.085]
Hybrid vs. DL	0.037	0.012	+ 5.1%	[1.6%, 8.9%]	[0.022, 0.062]
DL vs. Radiomics	0.032	0.041	+ 3.1%	[0.2%, 6.0%]	[0.011, 0.057]

The analysis confirmed that the hybrid model statistically outperformed both standalone models, particularly in terms of AUC and sensitivity, with differences reaching statistical significance (*p* < 0.05). These findings underscore the practical advantage of integrating radiomic and deep learning features into a unified predictive framework.

## Discussion

The results of the present study support the initial hypothesis that the integration of radiomic features and deep learning-derived representations from DCE-MRI significantly improves the accuracy of classifying luminal A and B subtypes of breast cancer. The proposed hybrid model demonstrated a clear advantage over models based solely on either feature type, showing superior performance on both internal and external validation cohorts.

DCE-MRI was selected as the primary imaging modality because luminal subtype differentiation is strongly associated with tumour vascularity and contrast enhancement kinetics, neither of which is fully captured by conventional non-contrast sequences. Although T1- and T2-weighted images were available for some patients, preliminary analyses showed that DCE-MRI-based models performed consistently better (ΔAUC ≈ 0.06–0.09). Therefore, these sequences were not included in the final hybrid framework. Future studies will explicitly evaluate the integration of multiple MRI sequences.

The high Dice Similarity Coefficient (0.892) achieved between two independent experts during semi-automated tumor segmentation confirms the reproducibility of contour delineation, which is a critical prerequisite for reliable feature extraction. The selected radiomic features encompassed both morphological attributes (e.g., Surface Volume Ratio) and textural characteristics (e.g., Entropy, Zone Variance), underscoring the complex spatial and intensity-based differences between luminal subtypes. The inclusion of these interpretable features enhanced the clinical relevance and transparency of the model.

Comparative analysis showed that the hybrid model achieved the highest performance metrics, including AUC = 0.921, sensitivity = 88.6%, and specificity = 89.7%. The AUC superiority over both the deep learning (DL) model and the radiomics model was statistically significant (*p* = 0.012 and *p* < 0.001, respectively). These differences remained consistent under bootstrapping and did not diminish during external validation, indicating strong model stability and generalizability. Achieving an AUC > 0.9 in a multicenter design, without tuning to specific scanners or institutions, highlights the scalability potential of the proposed framework.

SHAP analysis enabled the identification and quantification of the most informative features. Radiomic parameters such as GLCM_Entropy and Shape_Sphericity were among the highest contributors, alongside deep feature vectors extracted from the final layers of the 3D ResNet. This confirms that feature hybridization effectively captures both interpretable morphological patterns and abstract nonlinear dependencies not accessible through traditional methods. In addition, Grad-CAM visualizations demonstrated that the neural network focused on biologically relevant regions of the tumor, further enhancing clinician trust in model predictions.

The comparison of model performance also revealed an important practical insight: although the DL model outperformed the radiomics model in terms of AUC (0.884 vs. 0.852, *p* = 0.041), it did not reach the performance level of the hybrid model. This highlights the limitations of using deep features in isolation—particularly in studies with moderate sample sizes—despite prior model pretraining. Conversely, radiomics alone is constrained by its inability to capture the contextual and spatial complexity of tumor presentation, which is partially offset when deep features are incorporated.

External validation using an independent cohort from Bulgaria further confirmed the cross-site transferability of the hybrid model. The system maintained an AUC of 0.903 without the need for parameter recalibration, and the reduction in accuracy compared to the internal test set did not reach statistical significance. This is particularly relevant in the context of inter-institutional heterogeneity, which is typical in real-world clinical settings.

Thus, the combined approach not only improves classification accuracy but also enhances model interpretability, which is critical for clinical application in diagnosis and treatment planning. Rather than replacing existing radiological methods, it serves as a complementary tool, helping to overcome current limitations. The findings of this study may provide a foundation for future prospective research and the integration of such models into clinical workflows, with careful consideration of both ethical and technical aspects.

Limitations of this study include its retrospective design and the relatively limited size of the external validation cohort. In addition, although harmonized MRI and IHC protocols were followed, potential inter-center technical variability may have influenced the extracted features. Future research will aim to expand the cohort to include a more ethnically diverse population and to extend classification to additional molecular subtypes of breast cancer for a multi-class diagnostic framework.

Overall, the proposed hybrid strategy demonstrates high accuracy, robustness, and clinical applicability, effectively combining the strengths of radiomics and deep learning. These findings support the promise of multi-paradigm approaches in advancing personalized oncologic diagnostics.

In recent years, the challenge of accurately classifying molecular subtypes of breast cancer has been addressed with increasing sophistication through the application of radiomics and machine learning to MRI and mammography data. For example, the study by Rizzo [23] showed that radiomics-based models could effectively distinguish between benign and malignant lesions, as well as tumor subtypes. However, the ability to differentiate luminal A and B subtypes remained limited, with an AUC of only 74%.

In Zhou et al. [24], an interpretable XGBoost model based on multiparametric MRI achieved high accuracy in distinguishing luminal from non-luminal subtypes (AUC up to 0.956), but did not focus on the more granular classification between luminal subtypes. Similarly, studies [25] and [26] used radiomics-based methods incorporating tumor heterogeneity analysis and both intra- and peritumoral features, achieving AUC values of 0.93–0.95 for luminal vs. non-luminal classification, highlighting the value of integrating spatial characteristics of tumors.

Studies [27] and [28] further illustrate the potential of multimodal integration, combining data from mammography, MRI, and clinical records with deep learning to improve subtype classification and increase model interpretability. In Wang et al. [29] and Petrillo et al. [30], ensemble deep neural networks with automated tumor segmentation demonstrated high stability in classifying the four major molecular subtypes (AUC 0.68–0.84), but lower accuracy in distinguishing closely related luminal subtypes.

In Gaudio et al. [31], radiomics based on contrast-enhanced mammography achieved high accuracy (up to 94%) in classifying luminal and non-luminal histotypes. However, the study was limited by its exclusive focus on mammographic data. Both the review in Gaudio et al. [31] and the investigation in Gamal et al. [32] primarily addressed the assessment of response to neoadjuvant therapy using radiomics and artificial intelligence, an adjacent but distinct objective from the present study.

In contrast to Rizzo [23], where the accuracy of distinguishing luminal subtypes was limited (AUC = 0.74), our study achieved higher classification performance (AUC up to 0.92) through the integration of radiomic and deep learning features. Studies [24–27] applied radiomics-based models using multiparametric MRI, but did not incorporate hybrid approaches with 3D neural networks, and exhibited lower interpretability of the resulting models.

Unlike studies [28–30, 33, 34], which applied either deep learning or radiomics methods independently—often with limited accuracy and without the integration of clinical data—our study combines multiple data sources and demonstrates high model robustness in a multicenter setting. Furthermore, in contrast to the work in Gaudio et al. [31], which was constrained to contrast-enhanced mammography, we employed a comprehensive DCE-MRI protocol along with state-of-the-art interpretability techniques (SHAP and Grad-CAM), thereby enhancing the clinical trustworthiness of the model’s outputs.

## Conclusions

The present study demonstrated that the integration of radiomic features and deep features extracted from DCE-MRI using a 3D ResNet neural architecture significantly improves the accuracy of classifying luminal subtypes (A and B) of breast cancer, compared to using either approach independently.

Tumor segmentation showed high inter-observer reproducibility (Dice Similarity Coefficient = 0.892), ensuring the reliability of feature extraction. From a total of 107 radiomic parameters, 17 robust features were selected using LASSO regression, the majority of which reflected morphological and textural characteristics of tumors relevant to known pathophysiological processes.

A comparative analysis of three models (radiomics-only, deep learning-only, and hybrid) revealed that the hybrid approach achieved the best performance: AUC = 0.921, sensitivity = 88.6%, and specificity = 89.7%. This model significantly outperformed both alternatives in terms of AUC and accuracy (*p* < 0.05) and maintained consistent performance on an independent external validation cohort (AUC = 0.903, *p* = 0.21 vs. internal validation).

SHAP analysis showed that both radiomic features (e.g., GLCM Entropy, Sphericity, Skewness) and deep feature vectors extracted from the final layers of the 3D ResNet (e.g., DeepFeature_182) played a key role in the model’s decision-making process. Additionally, Grad-CAM attention visualization enabled localization of the tumor regions with the greatest predictive contribution.

Thus, the proposed hybrid model represents a promising tool for non-invasive molecular stratification of breast tumors and may serve as a foundation for personalized therapeutic strategies. The findings can support decision-making by multidisciplinary oncology teams, particularly in cases where IHC data are inconclusive or repeat biopsy is not feasible.

The model has potential for clinical application as a PACS-integrated software module or as a cloud-based service for automated analysis of DCE-MRI studies. Future work will focus on expanding the dataset, incorporating additional tumor subtypes, and conducting a prospective clinical trial to evaluate the model’s impact on patient outcomes.

Moreover, there are plans to integrate additional data modalities, including genomic and clinical-laboratory parameters, into a unified multi-omics predictive model to further enhance diagnostic precision and therapeutic personalization.

## Data Availability

All data generated or analysed during this study are included in this published article.

## Abbreviations

DCE-MRI: Dynamic contrast-enhanced magnetic resonance imaging
DL: Deep learning
IHC: Immunohistochemical
DSC: Dice Similarity Coefficient
